# Characterization of Esophageal Physiology Using Mechanical State Analysis

**DOI:** 10.3389/fnsys.2016.00010

**Published:** 2016-02-17

**Authors:** Richard E. Leibbrandt, Phil G. Dinning, Marcello Costa, Charles Cock, Lukasz Wiklendt, Guangsong Wang, Jan Tack, Dirk van Beckevoort, Nathalie Rommel, Taher I. Omari

**Affiliations:** ^1^Department of Human Physiology, School of Medicine, Flinders UniversityBedford Park, SA, Australia; ^2^Department of Gastroenterology and Hepatology, School of Medicine, Flinders UniversityBedford Park, SA, Australia; ^3^Department of Surgery, School of Medicine, Flinders UniversityBedford Park, SA, Australia; ^4^Gastroenterology, Neurogastroenterology and Motility, University Hospitals LeuvenLeuven, Belgium; ^5^Translational Research Center for Gastrointestinal Diseases (TARGID), University of LeuvenLeuven, Belgium; ^6^Department of Radiology, University Hospitals LeuvenLeuven, Belgium; ^7^Neurosciences, ExpORL, University of LeuvenLeuven, Belgium

**Keywords:** esophageal peristalsis, swallow, dysphagia, pressure, impedance, neural pathways

## Abstract

The esophagus functions to transport swallowed fluids and food from the pharynx to the stomach. The esophageal muscles governing bolus transport comprise circular striated muscle of the proximal esophagus and circular smooth muscle of the distal esophagus. Longitudinal smooth muscle contraction provides a mechanical advantage to bolus transit during circular smooth muscle contraction. Esophageal striated muscle is directly controlled by neural circuits originating in the central nervous system, resulting in coordinated contractions. In contrast, the esophageal smooth muscle is controlled by enteric circuits modulated by extrinsic central neural connections resulting in neural relaxation and contraction. The esophageal muscles are modulated by sensory information arising from within the lumen. Contraction or relaxation, which changes the diameter of the lumen, alters the intraluminal pressure and ultimately inhibits or promotes flow of content. This relationship that exists between the changes in diameter and concurrent changes in intraluminal pressure has been used previously to identify the “mechanical states” of the circular muscle; that is when the muscles are passively or actively, relaxing or contracting. Detecting these changes in the mechanical state of the muscle has been difficult and as the current interpretation of esophageal motility is based largely upon pressure measurement (manometry), subtle changes in the muscle function during peristalsis can be missed. We hypothesized that quantification of mechanical states of the esophageal circular muscles and the pressure-diameter properties that define them, would allow objective characterization of the mechanisms that govern esophageal peristalsis. To achieve this we analyzed barium swallows captured by simultaneous videofluoroscopy and pressure with impedance recording. From these data we demonstrated that intraluminal impedance measurements could be used to determine changes in the internal diameter of the lumen comparable with measurements from videofluoroscopy. Our data indicated that identification of mechanical state of esophageal muscle was simple to apply and revealed patterns consistent with the known neural inputs activating the different muscles during swallowing.

## Introduction

Digestion involves several steps, with appropriate mixing and propulsive movements along the digestive tract controlled by neurogenic and myogenic mechanisms. This results in the absorption of nutrients and water and eventually the formation and expulsion of waste products. The muscle of the digestive tract consists of an outer layer of longitudinal muscle surrounding an inner layer of circular muscle. The propulsion of gut content is mostly mediated through relaxation and contraction of the circular muscle, although it is likely that the longitudinal muscle has a secondary role to play. Within the small bowel and colon the neural architecture governing propulsion is characterized by polarized neural circuits comprising anally projecting inhibitory neurons and orally projecting excitatory neurons. Bayliss and Starling (1899) proposed that propulsion of the bolus is due to the activation of these polarized enteric pathways with oral contraction and anal relaxation.

Building upon this original concept our group has recently developed a *neuromechanical loop* hypothesis (Costa et al., 2013; Dinning et al., 2014). This hypothesis involves the activation of polarized enteric pathways by bolus distension (neuro-), with oral contraction and anal relaxation (-mechanical) resulting in propulsion of the bolus aborally to distend a new portion of the gut and initiate a new loop process. This propulsive activity becomes self-sustained, and is adaptable because the speed of propulsion is dependent upon the viscosity of content and size of the bolus (Costa et al., 2015).

The concept behind the neuromechanical loop is based upon a novel strategy which allows us to determine *the mechanical states of the muscle* during peristalsis. This is achieved by examining the relationships that exist between the changes in diameter with the corresponding changes in intraluminal pressures. By combining these two mechanical measurements we have established the existence of 12 mechanical states that describe when the gut is actively or passively contracting or relaxing or when it is in a state of quiescence. The calculation of the mechanical states has provided valued insight into factors that govern the movement of content (Costa et al., 2013; Dinning et al., 2014; Omari et al., 2015).

Application of the mechanical states to human gut motility may also help to provide insight into the physiological differences in gut function in health and disease. For example features of the motility of the esophagus can now be measured in great detail using *high-resolution manometry*. Manometric pressure-plot patterns in health and disease have been described for clinical diagnostic purposes, using the *Chicago Classification* (Kahrilas et al., 2015). However, to improve our ability to detect abnormalities in esophageal function in disease and to more clearly explain the pathophysiology of symptom generation, a better understanding of the neurogenic and passive mechanical factors that drive motor behaviors is still required.

The purpose of this study was to apply the technique of mechanical state analysis to recordings of the normal human esophagus during swallowing. We hypothesized that doing so would enable us to assess the major components of esophageal function, by allowing us to deduce when the muscle is actively contracting or relaxing in response to neural inputs as well as mechanical wall states which may stimulate sensory afferents, producing symptoms (The term “muscle” as used hereafter should be understood to refer to the circular muscle of the digestive tract, because of its dominant role during content propagation).

Calculation of the mechanical states requires accurate measurement of diameter. To approximate changes in diameter in a real-time and *in vivo* situation we have previously used intraluminal impedance, which is recorded in parallel with the manometry. In both *ex vivo* recordings in a rabbit colon (Costa et al., 2013) and *in vivo* recordings in the human pharynx and esophagus (Kim et al., 2014; Omari et al., 2015, 2016) it has been shown that intraluminal impedance measurements can be used to estimate changes in diameter in association with bolus movement, thus potentially negating the need for radiology. In this article, we provide further validation of this technique based on *in vivo* recordings of esophageal pressure, diameter and impedance.

Our study had three aims. Firstly, we aimed to use combined videofluoroscopy and esophageal manometry to characterize the mechanical state profile occurring within the distal esophagus based on the inter-relationships of luminal diameter (recorded by X-ray) and pressure change over time. Secondly, we aimed to determine if esophageal mechanical states can also be predicted using intraluminal impedance measurement to estimate diameter change in place of videofluoroscopy. In order to achieve this we sought to characterize the relationships that exist between changes in diameter and the corresponding changes in intraluminal impedance, hence enabling non-radiological application of the technique. Thirdly, following completion of the validation studies above, we aimed to apply the optimized pressure-impedance model to the entire esophageal body, examine regional differences in mechanical states along the esophageal body and compare these regional differences with the known mechanisms of esophageal integrative function.

## Materials and Methods

### Overview and Conceptual Framework for Defining Esophageal Mechanical States

In this study, the technique of video-impedance-manometry was used to record changes in esophageal luminal diameter and pressure. The changes in esophageal pressures, during the swallow of a barium-labeled bolus, were recorded by an indwelling impedance manometry catheter. The changes in the diameter of the distal esophageal lumen were measured using videofluoroscopy which visualized the passage of the bolus. These video images of each swallow were processed and converted into spatiotemporal diameter maps and these were temporally aligned in space and time with the corresponding pressure recording, creating pressure-diameter maps.

**Figure 1:**
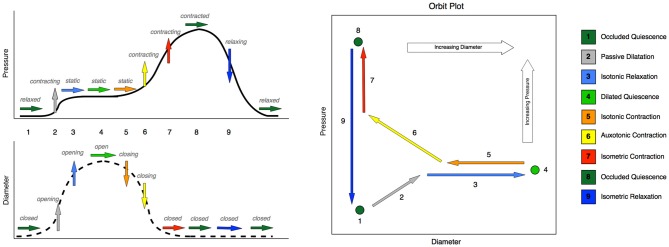
**Typical changes that occur in pressure and diameter in the esophageal body during swallowing, with the corresponding mechanical states.** Schematic time series plots for pressure and diameter are shown on the left-hand side, Number labels indicate time intervals during which changes occur in either diameter or pressure or both. Rightward-pointing arrows indicate no change in the time series data, while upward- and downward-pointing arrows indicate increases and decreases respectively. The color of the arrow indicates the mechanical state. The simultaneous relationship between pressure and diameter is shown on the right-hand side of the figure as a schematic orbit plot. Number labels indicate the same time intervals as in the time series plots. Arrows indicate the direction of change in pressure and diameter simultaneously. Note that in some instances of swallowing, the moderate pressure increase shown at step 2 does not occur, and instead pressure remains static over the entire period shown for steps 1 through 5. In such cases, state 2 (passive dilatation) would be absent, and the expected state sequence would proceed directly from 1 (occluded quiescence) to 3 (isotonic relaxation).

**Figure 2:**
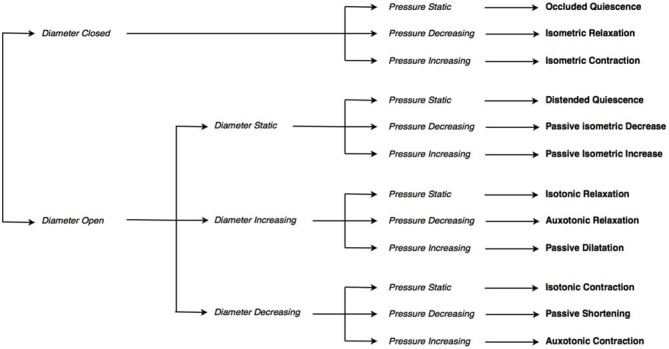
**The decision tree used to determine mechanical states.** The decision tree selects one of twelve possible mechanical states, based on: whether the diameter is open or closed, whether the diameter (if open) is static, increasing or decreasing, and whether pressure is static, increasing or decreasing.

By examining the relationships that exist between changes in diameter and the corresponding changes in pressure taken from the pressure diameter maps, the mechanical states of the muscle were determined (Costa et al., 2013). These mechanical states predict when the muscle is actively contracting or relaxing during periods of luminal occlusion or distension. During a human swallow these changes in diameter and pressure can be seen in Figure 1. The relationship between diameter and pressure over time can also be visualized by way of an “Orbit” plot (Figure 1). Previous studies examining peristalsis in the isolated rabbit distal colon in an organ bath have defined 12 possible mechanical states (eight active and four passive; Costa et al., 2013; Wiklendt et al., 2013; Dinning et al., 2014) and these have been detailed in Figure 2.

Once the mechanical states had been determined in the distal esophagus from the diameter pressure maps we assessed the accuracy of using impedance to calculate the cross sectional area of the lumen in the distal esophagus. To achieve this we found the correlation between the inverse measurement of impedance (called* admittance*, described below) and the corresponding video-measured diameter at each impedance segment level along the catheter array. We then calculated the mechanical states based upon admittance and pressure relationships and compared these findings to the mechanical states calculated from pressure and diameter relationships at each level. As video imaging was predominantly available for the distal esophagus only, validation against diameter was performed for distal esophagus only and then the pressure-impedance mechanical state model was applied to the recording of the entire esophageal body.

### Study Procedure

Nine healthy controls (5 male; 20–29 years, mean 23.4 years) were enrolled in the study. All subjects were screened and excluded if they had a history of gastroesophageal reflux disease, previous upper GI surgery, were taking medications known to impact on gastrointestinal motility or if they gave a history of oropharyngeal or esophageal dysphagia on pre-study interview. All subjects gave written informed consent prior to participation in the study. The study protocol was approved by the Research Ethics Committee, University Hospitals Leuven, Belgium.

Each subject was intubated with a 3.2 mm diameter solid state pressure and impedance catheter incorporating 36 1 cm-spaced pressure sensors and 16 adjoining impedance segments, each of 2 cm length (Unisensor USA Inc., Portsmouth, NH, USA). Subjects were transnasally intubated after topical anesthesia (lignocaine spray) and the catheter was positioned with sensors straddling the entire esophagus (upper esophageal sphincter, UES to stomach). Following accommodation to the catheter, a brief fluoroscopy screen ensured correct catheter position across the esophagogastric junction (EGJ). A standardized protocol comprising barium bolus swallows was captured simultaneously by continuous videofluoroscopy (25 frames/s) and the pressure-impedance acquisition system (data sampling at 20 Hz, Solar GI system, Medical Measurement Systems, Netherlands). The videofluoroscopy was positioned to capture the swallowed bolus moving through the distal esophagus and EGJ.

Three swallows of 5 ml liquid, semisolid and solid barium were recorded with the subjects sitting upright. Subjects were then changed to the decubitus body position and a further 5 ml liquid and semi-solid bolus were given. A solid bolus was not given to the subjects when they were lying down. Sodium chloride was added to all bolus material reducing the impedance to a level equivalent to normal 0.9% saline. A radiologist (Author DvB), assessed each swallow according to the classification system of Fox et al. (2004) using a seven-grade Likert-type scale to score bolus transport (1 = successful bolus transport, 7 = complete failure of transport). All swallows were classified as normal (score ≤ 2).

### Measurement of Esophageal Diameter Change During Swallow

For each of the swallows the location of the EGJ was identified on the video images. The pressure and impedance sensors proximal and distal to the EGJ were identified on the video images. As the impedance sensors were spaced at 2 cm intervals, the analysis was divided into 2 cm regions with pressure taken from the sensor located at the mid-point of the impedance segment.

The diameter was measured for each impedance segment in the distal esophagus based upon the simultaneously acquired videofluoroscopy images. These were digitally analyzed through the creation of spatiotemporal diameter maps. This was achieved using custom-written software in Matlab (MathWorks, Natick, MA, USA). Based upon work from our laboratory (Hennig et al., 1999; Costa et al., 2013) the diameter at each point along the length of the distal esophagus was calculated for each frame and converted into gray-scale pixels. Regions of minimal diameter (contraction) were represented on maps as white pixels, whereas maximal diameter (distension) was represented by black pixels. Additional custom written software, using Matlab, enabled measurements of the changes in diameters for each impedance segment. The relative changes in wall thickness with boluses of different diameters were not considered in this work.

**Figure 3:**
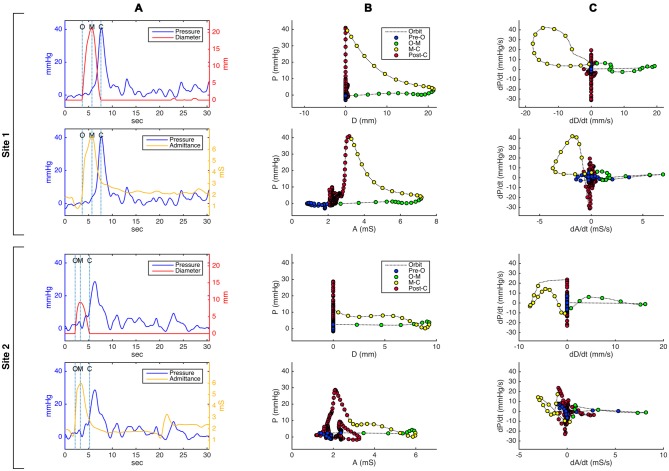
**Pressure-diameter and pressure-admittance relationships in the distal esophagus for an example swallow, shown as (A) temporally aligned time series data plots, (B) orbital plots of the raw data, and (C) orbital plots of the time derivative (gradient) of the data, for two different catheter sites.** Site 1 (top 2 rows in the figure) is located approximately 3 cm below the transition zone; site 2 (bottom 2 rows) is located approximately 8 cm below site 1 and 7 cm above the lower esophageal sphincter. Dotted vertical lines on the time series plots in **(A)** indicate key events related to the opening and closing of the lumen (O, luminal opening; M, maximal luminal extent; C, luminal closure). In the orbital plots in **(B)** and **(C)**, X time axes have been aligned in order to demonstrate differences and similarities between plots derived from admittance and plots derived from diameter. Circular markers on the orbit plots occur at regular time intervals of 0.15 s, and their color indicates the phase of luminal opening. The most salient visible difference between the diameter-based and admittance-based orbit plots is that admittance in some plots returns to a higher value post luminal closure compared to its pre-swallow baseline.

Representative plots of simultaneously recorded *diameter and pressure* or *admittance and pressure* measurements are shown in Figure 3. In order to create these, the diameter measurements were calibrated for magnification by using the known distance between visible adjacent sensors located on the catheter. The changes in esophageal diameters were expressed in relation to the net of the width of the indwelling catheter (~3.2 mm). Therefore when the lumen was fully closed on the catheter the diameter of the lumen was taken as 0 mm (lumen occlusive), rather than 3.2 mm. The diameter dataset was then smoothed using Gaussian smoothing with a time window of 0.25 s. The pressure and impedance data were exported from the acquisition system in comma-separated value text-file format, and smoothed using the same method as for the diameter. The impedance values were converted to *admittance* (inverse impedance; expressed in millisiemens, mS).

The space-time synchronized diameter, pressure and admittance data from multiple sites along the catheter array were then converted to spatiotemporal diameter, pressure and admittance maps. The diameter-admittance maps allowed us to assess the correlation between changes in diameter and changes in admittance. Diameter-pressure maps allowed us to establish the mechanical states of the muscles on the basis of changes in real diameter and the corresponding changes in pressure (see below). Pressure-admittance maps allowed for the identification of the mechanical states of the muscle in the absence of diameter measures from videofluoroscopy.

### Defining the Relationship Between Changes in Diameter and Changes in Admittance

The Pearson correlation coefficient (*r*) was used to assess the strength of temporal correlation between diameter and admittance, for each catheter location in each swallow individually.

In determining the correlation between these two variables, we focused specifically on their relative behavior during the phases of distension and occlusion. Hence Pearson’s correlation coefficient (*r*) between admittance and diameter was predominantly calculated for the main event of luminal opening and closing; this comprised over 80% of the analyzed data samples in all cases. Correlations were determined for every analyzable site for every swallow, this yielded a total of 282 correlation values.

### Definition of Mechanical States: Diameter-Pressure Method

Using the temporally aligned and synchronized diameter and pressure data (Figure 3A) at each location for each swallow, the relationship of pressure vs. diameter over time can be displayed as an “orbit plot” of the raw data (Figure 3B) or gradient data (i.e., time derivative data; Figure 3C). From these orbit plots, the 12 possible mechanical states were identified using a decision tree (see Figure 2). To apply the decision tree we optimized criteria for defining when the lumen opened or closed and if the lumen was open, whether the diameter was increasing, decreasing or remaining static. During a swallow event, the timings of first lumen opening, peak diameter and final lumen closure were determined using points of inflexion of the diameter curve. First lumen opening was defined when the gradient of positive inflexion reached 2 mm/s and final lumen closure was defined when the gradient of negative inflexion reached −2 mm/s. Following the identification of inflexion points, the 10th percentile of all gradients measured from opening to peak, and then from peak to closure, was taken as the threshold for increasing and decreasing diameter respectively. Across all sensors and all swallows, the mean threshold slope for increasing diameter was +1.09 mm/s, and −1.32 mm/s for decreasing diameter. Pressure increase and decrease were defined as exceeding thresholds of +10 mmHg/s and −10 mmHg/s respectively.

**Figure 4:**
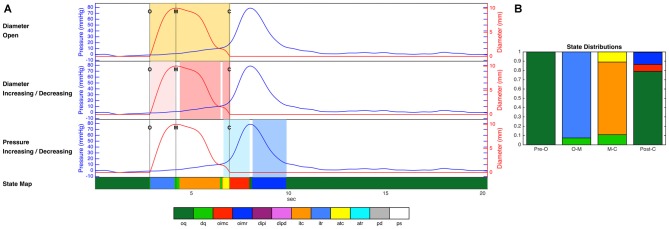
**(A)** Diameter and pressure time series for one example swallow, as well as the decisions regarding luminal opening, diameter change and pressure change used by the decision tree, and the final prediction of mechanical state over the whole time period (colored strip at the bottom of the figure). Dotted vertical lines on the time series plots **(A)** indicate key events related to the opening and closing of the lumen (O, luminal opening; M, maximal luminal extent; C, luminal closure). Orange shaded areas indicate the time period when the lumen is open. Light pink shading indicates diameter increasing, and darker pink indicates diameter decreasing. Light blue shading indicates pressure increasing, and darker blue indicates pressure decreasing. **(B)** The proportional distribution across states during the four time periods up until luminal opening (Pre-O), during luminal opening to maximal extent (O–M), during luminal closure (M–C), and after luminal closure (Post-C). The periods before and after the opening and closing of the lumen in these figures are dominated by occluded quiescence, while isotonic relaxation predominates during luminal opening, and isotonic and auxotonic contraction predominate during luminal closure. In addition, isometric contraction and relaxation are both present following luminal closure. Key to state labels: oq, Occluded Quiescence; dq, Distended Quiescence; oimc, Occluded Isometric Contraction; oimr, Occluded Isometric Relaxation; dipi, Diameter Increase + Pressure Increase; dipd, Diameter Increase + Pressure Decrease; itc, Isotonic Contraction; itr, Isotonic Relaxation; atc, Auxotonic Contraction; atr, Auxotonic Relaxation; pd, Passive Dilatation; ps, Passive Shortening.

The mechanical states can be seen for a single location for one example swallow in Figure 4. Data for each swallow were binned into four distinct time epochs, these were: (i) time period prior to opening; (ii) opening to maximal diameter/admittance; and (iii) maximal diameter/admittance to closure; and (iv) post swallow.

### Definition of Mechanical States: Admittance-Pressure Method

The mechanical state analysis procedure described above for the diameter-pressure data was repeated for the admittance-pressure data set, with admittance used to infer internal luminal diameter and diameter change. Following an iterative process to determine the optimal settings, the onset of luminal opening and luminal closure was defined using the admittance inflection point (gradient thresholds of +1 mS/s and −1 mS/s), and a slope of +0.57 mS/s and −0.57 mS/s defined whether admittance was increasing and decreasing. Figure 3 shows orbit plots for admittance and pressure, compared with those created from diameter and pressure at the same locations.

### Correspondence of Mechanical States Between Diameter-Based and Admittance-Based Methods

The degree of correspondence between the mechanical states as identified by the diameter-pressure model and the admittance-pressure model was then determined as the percentage of time samples for which the two methods produced exactly the same mechanical state. We also examine the overall proportional distribution of time spent in each of the mechanical states, for each of the two methods.

## Results

### Temporal Correlation of Luminal Diameter and Impedance

**Figure 5:**
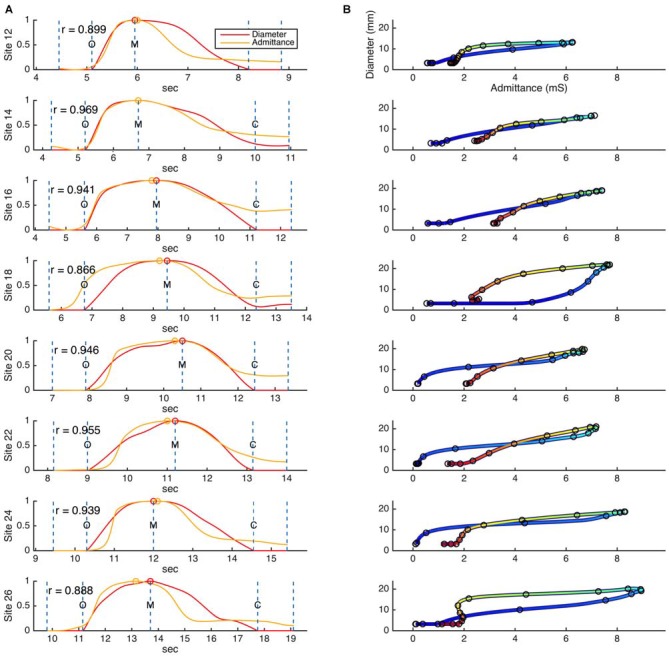
**(A)** Example time correlations and **(B)** orbit plots between diameter and admittance, as measured for a single swallow across eight different catheter sites in the distal esophagus. Dashed lines on the time correlation plots **(A)** indicate the beginning and end points of the correlation region, and key events related to the diameter data (O, luminal opening; M, maximal luminal extent; C, luminal closure). Circular markers indicate the maximum values of diameter and admittance. Color in the orbit plots **(B)** changes with time, from blue at the beginning of the correlation period to red at the end of the correlation period.

**Figure 6:**
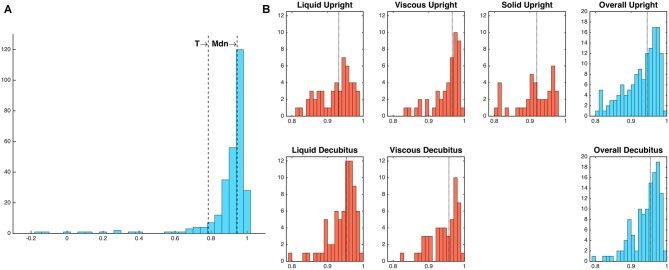
**(A)** Frequency distribution of diameter-admittance correlations (Pearson’s rho). Mdn, median; T, threshold value for outlying values (1.5 times the interquartile range below the lower quartile). **(B)** Frequency distributions of diameter-admittance correlations in relation to bolus type and body position (Note that a solid bolus was not administered in the decubitus position). Median values are indicated by vertical dotted lines. Twenty-four outlying values below threshold T have been omitted for ease of interpretability.

The relationships that exist between changes in diameter and changes in admittance in the distal esophagus are illustrated in Figure 5. Across all swallows the obtained Pearson correlation values between diameter and admittance were high (Mean = 0.896, Median = 0.945, SD = 0.163; Figure 6A). The distributions of the correlation values for bolus type and body position were as shown in Figure 6B.

### Predictions of Esophageal Mechanical States

When directly comparing the classification into mechanical states as identified by the two methods at each point in time, the two methods were found to identify the same mechanical state for 93.4% of all data samples.

**Figure 7:**
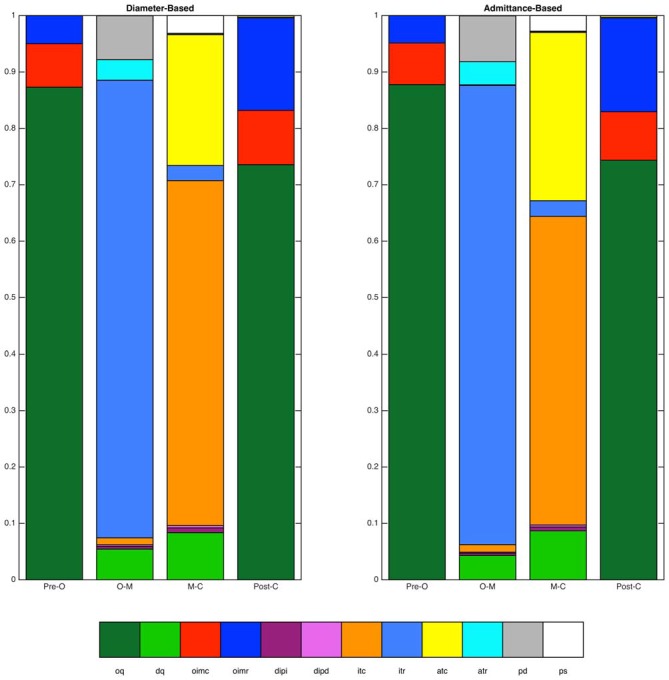
**Distributions of mechanical states identified by the diameter-based and admittance-based methods.** Distributions are shown over four time periods of interest (Pre-O, before luminal opening; O–M, luminal opening to maximal luminal extent; M–C, maximal luminal extent to luminal closure; Post-C, after luminal closure). Data are for the distal esophagus for all swallows combined. Overall agreement between the two methods was 93.4%.

Finally, the overall distributions of mechanical states for the diameter-based and admittance-based analyses, averaged across all catheter sites and all swallows, were calculated for each of the four time periods (Figure 7). No systematic differences in the overall proportions were apparent.

### Mechanical States in Relation to Bolus Type, Body Position and Esophageal Region

**Figure 8:**
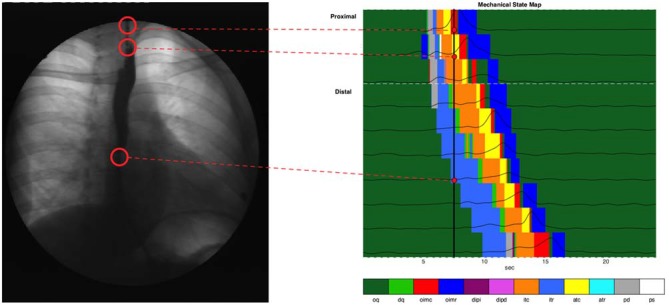
**Combined state map of an example swallow, showing mechanical state distributions for different regions.** Each horizontal colored strip represents the admittance-pressure-based state map at a single catheter location where impedance was recorded. Pressure time series plots at each location are overlaid on the state maps. Also shown is a black vertical line at the time of 7.5 s, and a videofluoroscopy image of the esophagus at that time, with corresponding anatomical sites linked to the catheter locations on the state map.

The admittance-pressure values allow construction of a *mechanical state map* for the entire esophageal body during bolus transport (Figure 8). In general, the mechanical states produced by our analysis were the ones that could be expected on the basis of the known behavior of pressure and diameter during swallowing, as shown in Figure 1, and occurred in the order predicted (apart from a number of minor, transient deviations, due to the inherent variability of diameter and pressure data). With propulsion of the bolus into the esophagus the proximal esophagus undergoes “passive dilatation” (Figure 8, shown in gray color), where increase in bolus pressure occurs simultaneously with an increase in diameter as the bolus enters each location in the proximal esophagus. This contrasts with what happens in the mid- and distal esophagus, where luminal opening is typically associated with isotonic relaxation (Figure 8, shown in light blue color), as the lumen opens with the arrival of the bolus and without any increase in pressure because the smooth muscle is relaxed by neural inhibition. Since the proximal esophagus contains striated muscle incapable of neural relaxation, the short period of isotonic relaxation that follows passive dilatation of the proximal esophagus (Figure 8), most likely reflects the fact that the UES and proximal esophagus below are drawn open during the swallow due to extrinsic mechanical factors such as anterior traction by the supra-hyoid muscles.

The active contraction of the esophagus which immediately follows passive distension and/or active relaxation are consistent with the predicted nature of peristalsis which, via isotonic and auxotonic active contraction (Figure 8, shown in orange and yellow), propels bolus contents distally and via isometric contraction (Figure 8, shown in red), seals the lumen proximal of the moving bolus preventing retrograde escape of luminal contents. Finally, isometric relaxation (Figure 8, dark blue) allows the lumen to return to its original quiescent state.

**Figure 9:**
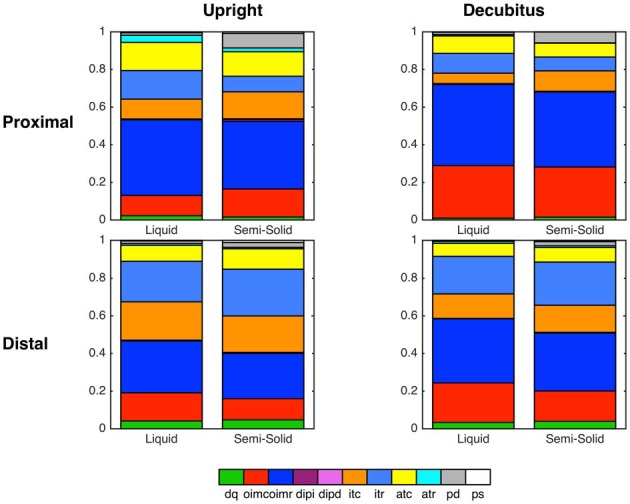
**Summary graphs based on all swallows showing state distributions across two esophageal regions and both body positions, separated according to bolus type (Liquid vs. Semi-Solid)**.

Summated data of mechanical states across all swallows for liquid and semisolid bolus type in both body positions (decubitus and upright) can be seen in Figure 9. In this figure, the esophagus has also been subdivided into the proximal esophagus (UES to transition zone) and the distal esophagus (transition zone to EGJ margin). The state of occluded quiescence has been omitted from this figure as it represents a background state of muscle inactivity and so is of less interest than the other states.

## Discussion

The current study was designed to apply the mechanical state analysis method, developed by our group in* ex vivo* recordings in animal colon (Costa et al., 2013) and validated in an *in vivo* human study of the UES (Omari et al., 2015, 2016), to investigate the changing mechanical states of the human esophageal body during swallowing. Our results show a high correlation between simultaneously recorded admittance and diameter in the esophagus, and support the notion that measured admittance can successfully be used in the place of measured diameter for our analyses. This is also corroborated by an acceptable level of correspondence between the mechanical states predicted using diameter or admittance. Having established the diameter-admittance correspondence we were able to apply the mechanical state analysis method to all of the esophageal body, whereby we were able to provide a detailed characterization of the pattern of active and passive muscular contractions and relaxations that occur during swallowed bolus transit.

Of the 12 mechanical states previously defined (Costa et al., 2013), eight were commonly seen associated with healthy swallowing. The sequence of these predicted and observed states (Figure 1 vs. Figure 8) was in accordance with known physiological mechanisms. The mechanical states that were observed in the animal colon but not the human esophagus included distended isometric increase and decrease, auxotonic relaxation and passive shortening. These absent mechanical states are all likely to be attributed to regions of the gut where outflow is restricted, resulting in dilation of the intestinal segment at the distal end. In a healthy human esophagus, with an unrestricted, normally relaxing lower esophageal sphincter, we would not expect such mechanical states to occur. Whether they occur in pathological conditions of outflow restriction of neurogenic (e.g., achalasia) or mechanical origin (e.g., eosinophilic esophagitis) remains to be determined.

In our current study, the pattern sequence of mechanical states was not substantially affected by bolus consistency or posture. However, the relative distribution of some states did change, most notably during the lumen opening phase where passive dilatation of the proximal esophagus became more prevalent with swallowing of semisolids and solids. We hypothesize that, in addition to the emergence of previously undetected states, the augmented presence of passive dilatation in the esophageal body may also prove to be a pathophysiological marker.

Furthermore, the ability to relate neuromechanical changes to symptoms such as the perception of dysphagia or esophageal pain, provides a framework for understanding the neurogenic and passive factors underpinning the generation of such esophageal symptoms. Such a conceptual framework, whereby the esophagus can have, at different times, active and passive roles in bolus transport and “switches back and forth” amongst roles under afferent sensory neural control has been previously proposed by Massey (1995). Mechanical state analysis of esophageal muscle has the potential to bring this important conceptual framework into clinical practice by providing a rationale for targeting existing or devising novel treatment strategies, based on the underlying neuromechanical changes.

**Figure 10:**
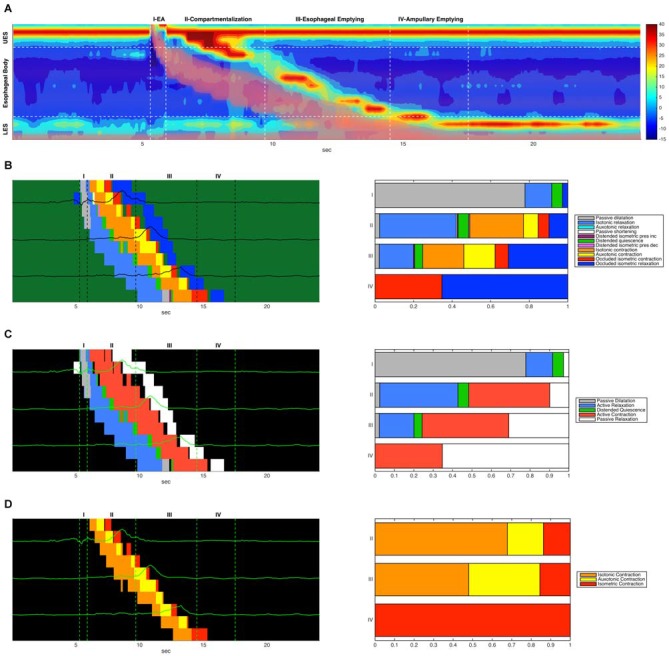
**(A)** Combined admittance-pressure topography for an example swallow. The presence of the bolus is indicated by pink shading in areas of low impedance. Also indicated are four time regions corresponding to the four phases of bolus transit described in Lin et al. (2014). **(B)** A combined mechanical state map (left) showing mechanical state distributions for different regions. Each horizontal colored strip represents the admittance-pressure-based state map at a single catheter location where impedance was recorded. Pressure time series plots at each location are overlaid on the state maps. Only the state map for the main esophageal body is shown. Also shown (right) is the proportional distribution of mechanical states that occurred in each of the four transit phases (occluded quiescence is omitted). **(C)** A corresponding simplified state map showing only active contractions and relaxations, passive dilatation and relaxation, and distended quiescence. **(D)** A corresponding state map that displays only the three forms of active esophageal contraction (isotonic, auxotonic and isometric).

Recently Lin et al. (2014) characterized esophageal bolus transport by defining four distinct phases: (1) accommodation of bolus contents propelled into the oral end of the esophageal lumen; (2) compartmentalized transfer of the bolus to the EGJ; (3) esophageal emptying of the bolus through the EGJ; and (4) a final phase of ampullary emptying, whereby the EGJ structures, separated during esophageal shortening, come back together. For comparison we have shown these four phases on a typical swallow and compared the analysis of Lin et al. (2014) with the mechanical states analysis performed in this article (Figure 10). In the proximal esophagus the mechanical states analysis reveals passive dilatation (gray region in Figure 10B) and this coincides with phase 1 from Lin et al. (2014). The phases 2–3 are characterized mainly by active contraction and relaxation of the smooth muscle in the distal esophagus (Figures 10B–D). The final phase (phase 4) is shown in the states map as predominantly active contractions (Figures 10B–D).

Previous studies suggest that muscle tension generated during the transition from a maximally distended lumen to an occluded lumen is augmented in patients who report dysphagia symptoms despite what appear to be “normal” esophageal motor patterns (Chen et al., 2013; Nguyen et al., 2013). As illustrated in Figure 10D, and also previously observed by Lin et al. (2014) there is a subtle “switch” of muscle state from isotonic to auxotonic contraction with the change from Phase 2 to Phase 3 transport (change in orange and yellow states from phase 2 to phase 3 in Figure 10D). Hence, during compartmentalized transport (Phase 2) muscle shortening occurs with no associated pressure increase, whilst during esophageal emptying (Phase 3) muscle shortening increasingly occurs in association with pressure increase. We believe that this subtle switch in contractile state (from isotonic to auxotonic) occurs as a consequence of flow resistance increasing at the EGJ, and we hypothesize that auxotonic contraction is yet another mechanical state which may increase in circumstances of EGJ outflow restriction.

The mechanical state methodology used here has some limitations which will still need to be refined to deal with potentially significant factors that affect the validity of the underlying measurements. For instance, distension due to air moving ahead of the bolus was invisible to our image analysis method and therefore not quantified. Air can also obscure the detection of luminal distension when using admittance. The effect of air was apparent in orbital plots of admittance vs. pressure, shown as a shift to the left at the time of bolus entry when compared to the corresponding diameter-pressure orbital plots (see the blue circular markers in central bottom image in Figure 3B). We also appreciate that the method of combined videofluoroscopy and automated image analysis used here to determine luminal diameter may be subject to a degree of inaccuracy due to image quality and radio-opacity of surrounding structures. However, our observations are largely in line with precise measurements of cross-sectional area using intraluminal ultrasound (Kim et al., 2014). It is however important to recognize that ultrasound only measures area at a focal point whilst the current method has the advantage of being able to simultaneously capture diameter changes over most of the axial length of the esophagus.

In conclusion, we have successfully applied the mechanical state analysis method to *in vivo* pressure-impedance recordings enabling the investigation of the changing mechanical states of the esophageal body during swallowing. The elucidation of switching of mechanical states in real-time and over space potentially allows deduction of efferent neural inputs and passive factors which promote and impede bolus flow. Further studies in dysphagia patients are warranted to examine esophageal mechanical states in relation to pathological flow associated with symptom generation.

## Author Contributions

REL: analysis and interpretation of data, draft and critical revision of manuscript. PGD, TIO: study concept and design, interpretation of data, draft and critical revision of manuscript. LW, GW: analysis and interpretation of data, critical revision of manuscript. MC, CC: interpretation of data, critical revision of manuscript. JT, DvB, NR: data acquisition, interpretation of data, critical revision of manuscript.

## Conflict of Interest Statement

The authors declare that the research was conducted in the absence of any commercial or financial relationships that could be construed as a potential conflict of interest.
